# Gene Expression Profiling Analysis Reveals Putative Phytochemotherapeutic Target for Castration-Resistant Prostate Cancer

**DOI:** 10.3389/fonc.2019.00714

**Published:** 2019-08-02

**Authors:** Solomon Oladapo Rotimi, Oluwakemi Anuoluwapo Rotimi, Abdulkadir Ayo Salako, Paul Jibrin, Jelili Oyelade, Emeka E. J. Iweala

**Affiliations:** ^1^Department of Biochemistry and Molecular Biology Research Laboratory, Covenant University, Ota, Nigeria; ^2^Department of Surgery, Obafemi Awolowo University, Ile-Ife, Nigeria; ^3^Department of Pathology, National Hospital, Abuja, Nigeria; ^4^Department of Computer and Information Sciences, Covenant University, Ota, Nigeria

**Keywords:** castration-resistant, prostate cancer, phytochemicals, soluble guanylate cyclase, gene expression data

## Abstract

Prostate cancer is the leading cause of cancer death among men globally, with castration development resistant contributing significantly to treatment failure and death. By analyzing the differentially expressed genes between castration-induced regression nadir and castration-resistant regrowth of the prostate, we identified soluble guanylate cyclase 1 subunit alpha as biologically significant to driving castration-resistant prostate cancer. A virtual screening of the modeled protein against 242 experimentally-validated anti-prostate cancer phytochemicals revealed potential drug inhibitors. Although, the identified four non-synonymous somatic point mutations of the human soluble guanylate cyclase 1 gene could alter its form and ligand binding ability, our analysis identified compounds that could effectively inhibit the mutants together with wild-type. Of the identified phytochemicals, (8′R)-neochrome and (8′S)-neochrome derived from the Spinach (*Spinacia oleracea*) showed the highest binding energies against the wild and mutant proteins. Our results identified the neochromes and other phytochemicals as leads in pharmacotherapy and as nutraceuticals in management and prevention of castration-resistance prostate cancers.

## Introduction

Malignancy of the prostate is the most commonly diagnosed cancer in men worldwide and ranked second as the cause of death, in cancer-related diseases (1). The burden of this disease is on the black population, who have one in four chance of getting the disease in their lifetime (2, 3). Hence, being black is a major risk factor for this disease and accounts for the disparity in the risk and outcome of the disease. Recent data suggest that the developing countries experience, and may continue to experience, disproportionate morbidity and mortality of this disease (4).

The development of both normal and malignant prostatic cells, as well as the proliferation of advanced carcinoma of the prostate, is highly dependent on androgens (5). Therefore, it has been hypothesized that androgens play a causal role in prostate tumorigenesis. Consequently, the primary therapeutic goal for prostate cancer is to reduce the levels of androgen (5). This is achieved through a group of treatment called androgen deprivation therapy. This treatment is universally accepted as the first line of treatment for prostate cancer, and it can be achieved either pharmacologically (chemical castration) or surgically (orchiectomy) (6). Despite these treatment options, a complication associated with a resurgence of androgen and elevation of prostate-specific antigen (PSA), arises toward the late stage of the disease. This state is referred to as castration-resistant prostate cancer (CRPC), and it is characterized by loss of ability to respond to androgen deprivation therapy and the recurrence of prostate cancer and subsequent metastasis (7). This recurrence of disease may occur in up to 40% of the patients (8, 9). For example, Bello (9) reported that 48 of 161 prostate cancer patients in Sub-Saharan Africa treated with androgen deprivation therapy developed CRPC, over a period of 4 years. Hence, CRPC continues to make prostate cancer the leading source of cancer mortality for men, particularly among the black race (4).

The transformation of hormone-dependent prostate cancer cells to castration-resistant ones is largely driven by upregulation of the activity of androgen receptor (10). This upregulation is often a consequence of: (1) the overexpression of the androgen receptor, which is observed in 22–30% of CRPC (11), (2) gain-of-function mutation of androgen receptor gene which occurs more often in 10–30% CRPC patients (12, 13), or (3) metabolic changes to the source of intratumoral dihydrotestosterone, as reviewed by Sharifi (14). Despite the cocktail of drugs targeting these well-established mechanisms, most of the pharmacological agents are ineffective against CRPC.

The limited treatment options for CRPC include secondary hormonal manipulations (with agents such as diethylstilbestrol, cyproterone and megestrol acetate), radiotherapy (with radium 223) (7, 15), drugs (such as docetaxel-which was approved by US Food and Drug Administration due to survival benefit only) (16) and novel drugs (like abiraterone, which has shown more promise in men of African ancestry in clinical trial) (17). Hence, CRPC continues to be a major factor contributing to the high mortality rate of prostate cancer, particularly in low resource countries, where the incidence of the disease is on the rise (3). In order to understand the factors that underlie the development of CRPC, previous studies have employed differential gene expression analysis to characterize the genetic and molecular factors that drive a prostate cell into being resistant to castration (18). It is therefore expedient to explore the available data in identifying novel targets, and subsequently, putative therapeutics for this disease in order to improve clinical outcomes and increase survival.

One significant and continuous source of novel drug-leads is the medicinal plants (19, 20). Phytochemicals derived from medicinal plants are structurally complex and diverse. Furthermore, many phytochemicals have been reported to possess cytotoxic properties and potentially useful as anti-cancer agents (21, 22). For example, Taxol, derived from Pacific yew tree (*Taxus brevifolia*) has been a success story and remains the best-selling anticancer drug for the treatment of ovarian cancer, breast cancer, and non-small cell lung cancer for up to four decades (20), as well as gastroesophageal, endometrial, cervical, prostate, and head and neck cancers (23). Also, Omacetaxine mepesuccinate originally derived from bark extracts of *Cephalotaxus harringtonii* and *Cephalotaxus fortune* with the trade name Synribo®, is used for the chronic myeloid leukemia (22). The success story has led to the clinical trials of over 100 natural products or natural product-derived compounds, the majority of which are on cancer treatment (24). Although a derivative of Toxol, Cabazitaxel®, is now in phase III clinical trial for the CRPC, the extension of life expectancy has only been by 3 months (25). Yet more phytochemicals have been suggested to be useful as preventive nutraceuticals and/or neo-adjuvant for prostate cancer in diverse populations (26, 27).

There is, therefore, need to use reverse pharmacology approach in developing the treatment for CRPC (28). To achieve this, this study analyzed the differentially expressed genes that drive CRPC and identified novel drug targets, as well as putative phytochemicals that can serve as inhibitors for the identified targets and its somatic variants.

## Materials and Methods

### Derivation of Microarray Data

The gene expression profile of GSE21887 (https://www.ncbi.nlm.nih.gov/geo/query/acc.cgi?acc=GSE21887) (18) was obtained from Gene expression omnibus (GEO) of the National Center for Biotechnology Information (NCBI). GSE21887 was based on GPL570 [HG-U133_Plus_2] Affymetrix Human Genome U133 Plus 2.0 Array. These data were derived from a xenograft model of prostate cancer, KUCaP-2, expressing wild-type androgen receptor and producing PSA. In order to identify the genes that drive the proliferation of prostate cancer cell following castration, we extracted data from eight chips for further analysis. These chips represented four samples of castration-induced regression nadir (GSM544233, GSM544234, GSM544235, and GSM544236) and compared with four samples of castration-resistant regrowth (GSM544237, GSM544238, GSM544239, and GSM544240).

### Differential Gene Expression Analysis

The derived raw Affymetrix expression data were initially pre-processed and normalized and then analyzed to identify the differentially expressed genes using Limma package in R language (29). First, the raw data from the probe set were summarized by calculating the expression values for the probe set using Microarray Suite 5.0 (MAS5, the standard Affymetrix algorithm) in R (30, 31). Furthermore, we used the linear regression model in Limma package to compare the castration-induced regression nadir samples and castration-resistant regrowth samples. Only the genes with |logFC| > 2.0 and the *p* < 0.01 were chosen as differentially expressed genes. Out of the list of the differentially expressed genes, we considered the gene with the highest fold change and lowest *p*-value for further analysis. Hence, we carried out further analysis on the human soluble guanylate cyclase 1 subunit alpha 2 (GUCY1A2).

### Identification of Gene Variants

Human genes are often highly polymorphic, and protein mutants determine the outcome of therapy.

In order to identify the somatic genetic variants of human GUCY1A2 in prostate cancer, we downloaded its missense mutation data from the Genomic Data Commons (GDC) Portal of National Cancer Institute (https://portal.gdc.cancer.gov/) (32). The missense mutation data from GDC was downloaded with Variant Effect Predictor (VEP) (33), SIFT (34), and PolyPhen (35) results.

### Homologous Modeling of Human GUCY1A2

The amino acid sequence of wild-type human GUCY1A2 (Uniprot ID: P33402) was retrieved from the UniProt database (http://www.uniprot.org). This protein sequence was used for predicting the 3D structure of the wild and mutant human GUCY1A2 using SWISS-MODEL (https://swissmodel.expasy.org/) (36). SWISS-MODEL is a fully automated server for predicting the 3D structure of proteins using the crystal structure of the similar protein as the template. For this purpose, we used human guanylate cyclase soluble subunit alpha-3 (pdb ID: 3uvj.1.A) as a template.

### Functional Consequence of the Missense Mutations on GUCY1A2

The effect of the mutations on the stability of human GUCY1A2 protein was assessed using I-Mutant adaptation 2.0. I-Mutant is an internet support vector that evaluates mutation prompted adjustments in protein dependability (37). It estimates the free energy changes value (DDG) as the difference between the unfolding Gibbs free energy value (DG) for the wild-type protein and that of the mutant protein (DDG or DDG = DG mutant – DG wild-type). Potential (surge or reduction) in the DDG is also predicted, along with a reliability index (RI) for the results, where the lowest and highest reliability are 0 and 10, respectively (38). Meanwhile, project HOPE (www.cmbi.ru.nl/hope/) (39) and MutPred (http://mutpred.mutdb.org/) (40) were used to identify the structural and functional consequences of the mutations on the human GUCY1A2 protein.

### Virtual Screening of Phytochemicals Against Human GUCY1A2 Variants

Structural data of 242 experimentally-validated naturally occurring anti-prostate cancer compounds were obtained from Naturally Occurring Plant-based Anti-cancer Compound-Activity-Target (NPACT) dataset (http://crdd.osdd.net/raghava/npact/index.html) (41). The compounds and those of the protein variants were imported into Molegro Virtual Docker (MDV). MDV was used for structural optimization and virtual screening as earlier described (42).

### Absorption, Distribution, Metabolism, and Excretion (ADME) Assessment of the Lead Phytochemicals

Characteristics of theoretical ADME and toxicological effects of the phytochemicals were determined by *in silico* analysis, using the SwissADME software (43). SwissADME is an online computational tool that also allows the prediction of the following pharmacokinetic characteristics: gastrointestinal absorption (GI), P-glycoprotein (P-gp) substrate, the inhibitor of some cytochromes P450 (CYP) known to be regularly involved in the interactions with xenobiotics (CYP1A2, CYP2C19, CYP2C9, CYP2D6, and CYP3A423) and blood-brain barrier permeant (BBBP).

## Results and Discussion

**Figure 1:**
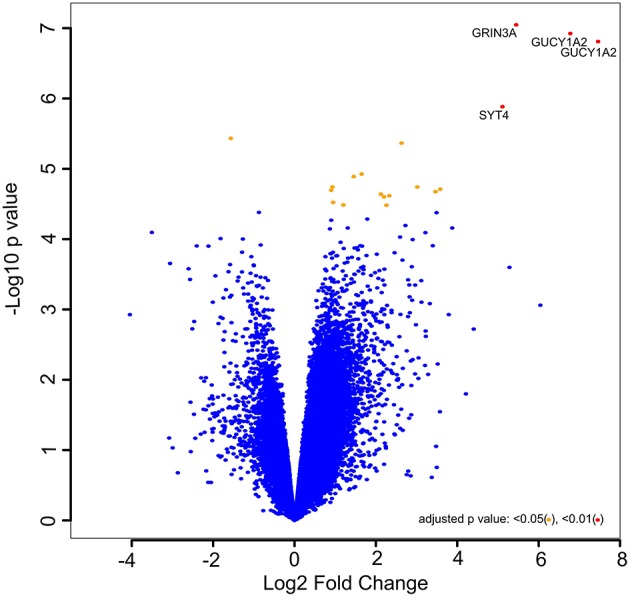
Volcano plot of –log10 (*p* values) vs. log2 fold change. The –log10 (*p* values) represents the level of significance of each gene while log2 fold change represents the difference between the levels of expression for each gene between the castration-induced regression nadir and castration-resistant regrowth groups.

The normalized Affymetrix data were used to determine the biological significance of each gene in driving castration-induced regression of prostate cancer into castration-resistant regrowth. The results for genes with |logFC| > 2.0 and the *p* < 0.01 are presented in Table S1. Meanwhile, Figure 1 represents the volcano plot of the distribution of the level of expression of genes not just according to statistical significance but also biological significance, as demonstrated by fold change. The genes represented by points at the upper far right of the graph are those considered to be significantly important in driving the castration responsive prostate cancer cells into castration resistance. The analysis showed that GUCY1A2, GRIN3A, and SYT4 are the most biologically important genes involved in the pathogenesis of CRPC in this patient-derived xenograft model. This differential expression analysis identified GUCY1A2, as the most significantly upregulated gene and biologically important in driving prostate cancer from castration-induced regression to castration-resistant growth. Hence, it was selected as the putative drug target for virtual screening. This gene codes for one of the peptides that make up soluble guanylyl cyclase (sGC) (44). sGC is a heterodimeric hemoprotein that is made up of two alpha and two beta subunits and serves as the intracellular receptor for nitric oxide. It mediates the biological function of nitric oxide, resulting in the formation of 3′, 5′-cyclic guanosine monophosphate and activation of protein kinase G (45). However, the alpha subunit of this protein complex has now been recognized to be regulated by the androgen receptor, in a non-nitric oxide-dependent mechanism, to mediate the growth of prostate cancer, both in the presence or absence of physiological concentration of androgen (46). Cai et al. (46) further reported an elevated level of expression of the alpha subunit of sGC in hormone-refractory prostate cancer at both mRNA level and protein (47). This is consistent with the immunohistological data in the Human Protein Atlas (48), that show the localization and elevated expression of this protein at the cytoplasmic/membranous nuclear in high-grade prostate adenocarcinoma. A major mechanism by which sGCα promotes prostate cancer is by associating with and sequestering p53 in the cytoplasm, leading to suppression of apoptosis (46). This observation strongly suggests that sGCα is a drug-able target for CRPC.

Interestingly, valproic acid, an anticonvulsant derived from *Valeriana officinalis* and shown to repress the expression of sGCα mRNA, has been reported by previous studies and a clinical trial to be useful in treating CRPC (49). However, this effect has solely been attributed to its histone deacetylases-inhibitory property (50, 51), without considering its anti-GUCY1A2 property. It is, therefore, possible that the suppression of GUCY1A2 is a complementary mechanism that was not explored by these previous investigators.

Although previous studies (44, 46, 52) have identified sGCα as a drug-able target for prostate cancer, our investigation represents the first to single it out as a major drug target, particularly, for CRPC. Again, these previous efforts have been directed at developing novel peptide that targets and inhibits sGCα activity (44, 52). However, therapeutic peptides have some significant drawbacks which include low membrane permeability, poor stability, and short half-life (53).

Since plant-derived chemicals have served, and continue to serve, as major sources for cancer chemotherapeutic and chemopreventive agents from time immemorial, we, therefore, exploited this vast resource of experimentally determined anti-prostate cancer phytochemicals to identify compounds or leads that could inhibit this protein.

The 242 plant-derived natural compounds with experimentally determined anti-prostate cancer activity were downloaded from NPACT database (41). The molecular docking results of the top 20 of these compounds are presented in Table 1. Also presented in the table is the chemical class of the compounds, the sources, and chemical structures. The highest number of the compounds are terpenoids with (8′R)-neochrome being the putatively most active compound. The binding energy and parameters of these compounds compared with methylene blue showed that all 20 phytochemicals have stronger binding energy than methylene blue (Table 2). However, the structural alterations induced by the somatic mutations alter the binding of these ligands. The compounds investigated have been experimentally validated for anti-prostate cancer activity, and because some are from edible plants, they may serve as neo-adjuvants or nutraceuticals in the prevention of CRPC (26). It is worthy of note that the phytochemicals we selected have higher binding energy to the modeled GUCY1A2 protein than methylene blue-which has been approved a pharmaceutical antagonist of guanylate cyclase (54). (8′R)-Neochrome being the compound with the higher binding energy −160.75 Kcal/mol, followed by (8′S)-neochrome (−152.102 Kcal/mol), 22-epicalamistrin (−151.131 Kcal/mol), 3-beta-O-(E)-feruloylbetulin (−145.363 Kcal/mol), 3-hydroxy-6′-desmethyl-9-O-methylthalifaboramine (−140.511 Kcal/mol) and Aculeatin A (−142.851 Kcal/mol).

**Table 1:** Compounds with highest binding energies against GUCY1A2 protein.

**Compound name**	**Class**	**Source**	**Structure**
(8′R)-neochrome	Terpenoids	*Spinacia oleracea*	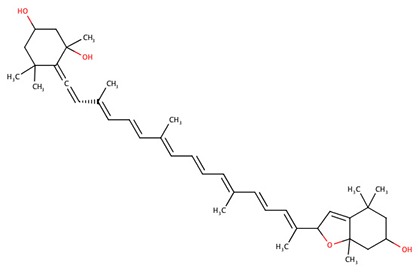
(8′S)-neochrome	Terpenoids	*Spinacia oleracea*	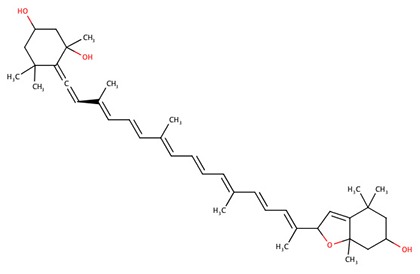
22-epicalamistrin	Polyketides	*Ampelocissus sp*	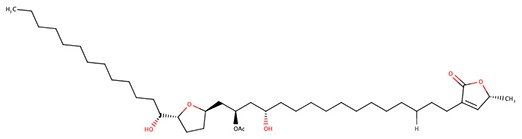
3-beta-O-(E)-feruloylbetulin	Terpenoids	*Celtis philippinensis*	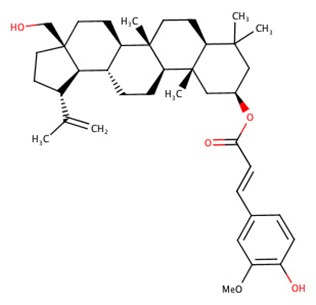
3-hydroxy-6′-desmethyl-9-O-methylthalifaboramine	Alkaloids	*Thalictrum faberi*	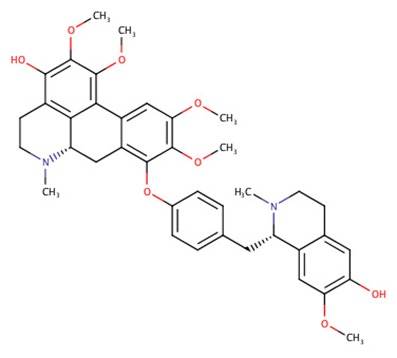
Aculeatin A	Polycyclic aromatic	*Toddalia asiatica*	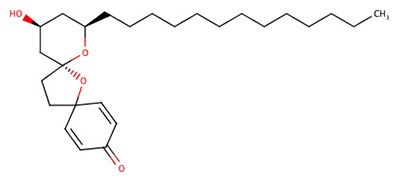
Annoglaucin	Polyketides	*Annona glauca*	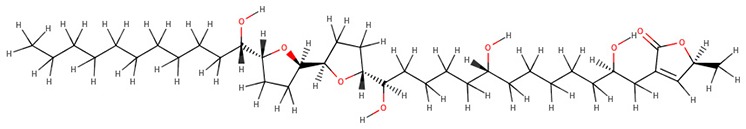
Bullatetrocin	Polyketides	*Asimina triloba*	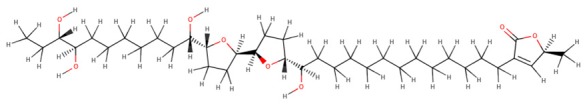
cis-3-O-p-Hydroxycinnamoyl Ursolic Acid	Terpenoids	*Vaccinium macrocarpon*	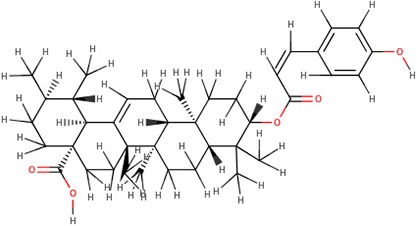
Desacetyluvaricin	Polyketides	*Uvaria accuminata*	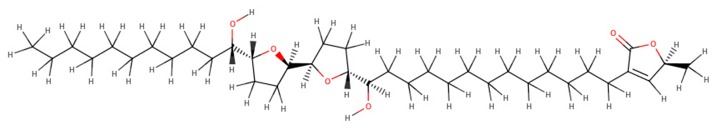
Foveoglin A	Benzofuranoids	*Aglaia foveolata*	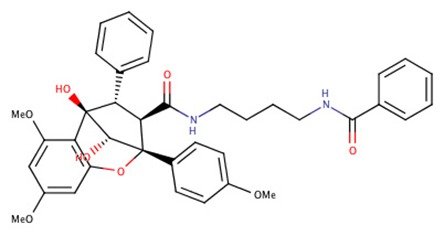
Foveoglin B	Benzofuranoids	*Aglaia foveolata*	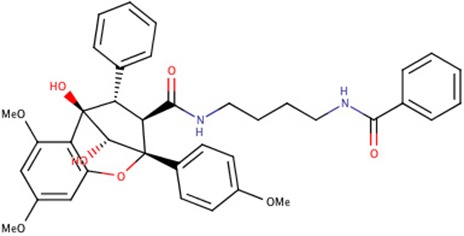
Longimicins A	Polyketides	*Asimina longifolia*	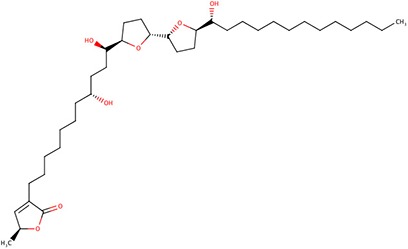
Melianin B	Terpenoids	*Melia volkensii*	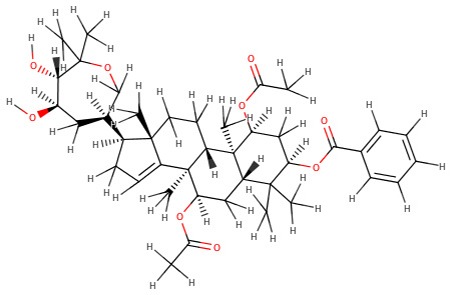
Melianin C	Terpenoids	*Melia volkensii*	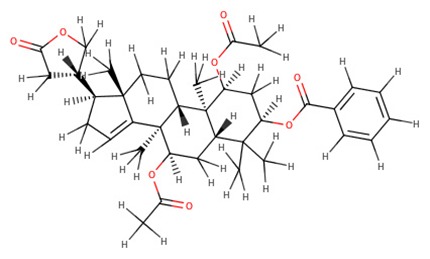
Meliavolkinin	Terpenoids	*Melia volkensii*	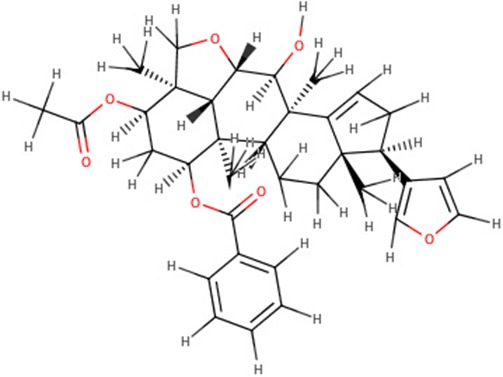
Muricatetrocin C	Polyketides	*Rollinia mucosa*	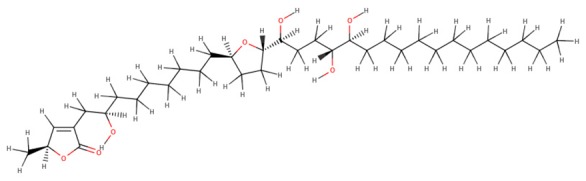
Rollidecin A	Polyketides	*Rohia emorginoto*	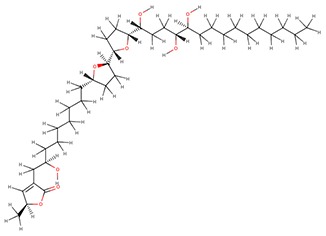
Silvestrol	Terpenoids	*Aglaia foveolata*	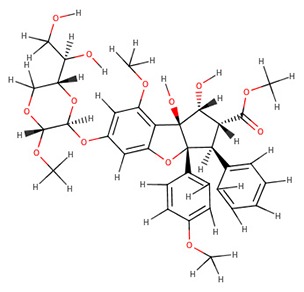
Vinblastine	Alkaloids	*Catharanthus roseus*	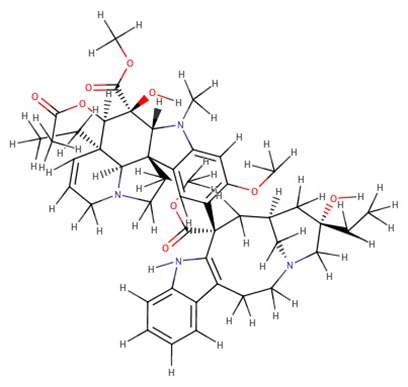

**Table 2:** Biding energies, molecular weight, and targets of anti- GUCY1A2 phytochemicals.

**Compound name**	**MolDock score**	**Molecular weight (g/mol)**	**Target**
Methylene blue	−67.2156	319.85	Wild
(8′R)-neochrome	−160.75	601.88	Wild, G723S, Q217H, P676L
(8′S)-neochrome	−152.102	601.88	Wild, G723S, Q217H, P676L
22-epicalamistrin	−151.131	593.43	Wild, G723S, R381Q
3-beta-O-(E)-feruloylbetulin	−145.363	604.86	Wild, G723S, Q217H, P676L
3-hydroxy-6′-desmethyl-9-O-methylthalifaboramine	−140.511	667.77	Wild, G723S, P676L
Aculeatin A	−142.851	418.61	Wild
Annoglaucin	−138.378	638.92	Wild, R381Q
Bullatetrocin	−137.773	638.92	Wild
cis-3-O-p-Hydroxycinnamoyl Ursolic Acid	−138.318	602.84	Wild, G723S
Desacetyluvaricin	−144.891	606.92	Wild
Foveoglin A	−142.354	650.72	Wild, G723S, Q217H, R381Q
Foveoglin B	−144.479	650.72	Wild, G723S, R381Q, P676L
Longimicins A	−151.925	622.92	Wild, G723S, R381Q, P676L
Melianin B	−137.738	694.89	Wild, Q217H
Melianin C	−137.917	620.77	Wild, G723S, Q217H, R381Q
Meliavolkinin	−153.697	574.7	Wild, G723S, Q217H, R381Q
Muricatetrocin C	−137.563	596.88	Wild P676L
Rollidecin A	−138.529	638.92	Wild, G723S
Silvestrol	−156.426	654.66	Wild, G723S, Q217H, R381Q
Vinblastine	−141.78	810.97	wild, G723S, Q217H, R381Q

The somatic mutations of GUCY1A2 that have been experimentally recorded in the prostate cancer were retrieved from TCGA and presented in Table 3. The mutations are R381Q, G723S, Q217H, and P676L. Polyphen recognizes all mutations as probably damaging while only R381Q is considered tolerated following SIFT analysis. However, as illustrated in Table 4, all the mutations could alter the stability of the protein, with P676L increasing the stability but with a low reliability index. Furthermore, functional analysis of the structural impact of these mutations predicted substantial alterations, not just in the function but also, in the ability of the protein to bind ligand (Table 5). This has clinical and pharmacological implications because such mutations now constitute a major problem resulting in the reduction of the efficacy of cancer chemotherapy. In order to account for this, we studied the effects of reported non-synonymous somatic mutation in GUCY1A2 on its protein structure and function. We further investigated the alterations in the binding energy between the phytochemicals and the mutant GUCY1A2 proteins. The phytochemicals have varying degrees of preference for different forms of this protein. We observed that none of the phytochemical could effectively bind all the mutant protein. (8′R)-neochrome, (8′S)-neochrome, 3-beta-O-(E)-feruloylbetulin, Foveoglin A, Foveoglin B, Longimicins A, Melianin C, Meliavolkinin, Silvestrol, and Vinblastine were able to effectively bind three of the four mutant protein, with the neochromes having the highest binding energy (Table 2). This phenomenon has been reported in different cancer drug-receptor interaction and often results in the reduction of drug efficiency or drug resistance and an important factor in drug pharmacogenomics (55). For example, somatic mutations in the aromatase gene CYP19 alter the efficacy of aromatase inhibitors when used as neoadjuvant therapy for breast cancer (56), while T790M mutation (rs121434569) reduces the effectiveness of Epidermal growth factor receptor inhibitors in treating lung adenocarcinomas (57). It is therefore of clinical importance to consider the somatic mutations in the drug receptors and target in the drug development process. Our results showed that (8′R/S)-Neochromes have a high binding affinity for all the variants of GUCY1A2 protein. (8′R/S)-Neochromes are neoxanthins that are naturally derived from vegetables such as spinach (*Spinacia oleracea*) during gastrointestinal digestion (58). Previous studies have reported the cytotoxic effects of Neochromes and their contribution to the anticancer effect of spinach (59, 60). Precisely, Kotake-Nara et al. (61) reported it to be cytotoxic against PC-3 human prostate cancer cells with IC_50_ of 1.2 μMol/L, and it has been suggested that p53 plays a minimal role in its mechanism of cytotoxicity (62). Although p53 protein is critical to cancer therapy due to its universal inactivation in human malignancies, the observation of Kotake-Nara et al. (61) and that of Cai et al. (46) imply that (8′R/S)-Neochrome may be potent in inducing apoptosis in cancer cells with or without p53 inactivation.

**Table 3:** Impact of somatic functional mutations on GUCY1A2.

**Mutation**	**Impact**		
	**VEP**	**SIFT**	**PolyPhen**
R381Q	Moderate	Tolerated	Probably damaging
G723S	Moderate	Deleterious (low confidence)	Probably damaging
Q217H	Moderate	Deleterious	Probably damaging
P676L	Moderate	Deleterious	Probably damaging

**Table 4:** Effects of mutation on stability of GUCY1A2 protein.

**Mutation**	**Stability**	**Reliability index**	**DDG (Kcal/mol)**
R381Q	Decrease	8	−1.32
G723S	Decrease	8	−0.97
Q217H	Decrease	6	−0.62
P676L	Increase	1	1.17

**Table 5:** Schematic structures of the original (left) and mutant (right) amino acid for each mutation with the functional consequence.

**Mutation**	**Structure**	**Functional consequence**
R381Q	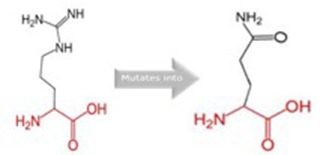	The mutant residue is smaller, positively charged, and located in a domain that is important for the main activity of the protein. Hence, mutation of the residue might disturb the function. The change in net charge can cause loss of interactions with other molecules or residues.
G723S	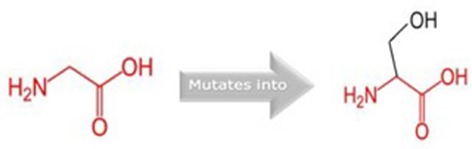	The mutant residue is bigger than the wild-type residue. The wild-type residue is a glycine, the most flexible of all residues. This flexibility might be necessary for the protein's function and mutation of this glycine can abolish this function. This mutation will force the local backbone into an incorrect conformation and will disturb the local structure.
Q217H	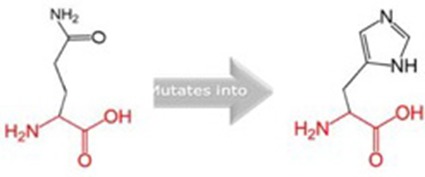	The mutant residue is bigger than the wild-type residue and located in a domain that is important for binding of other molecules.
P676L	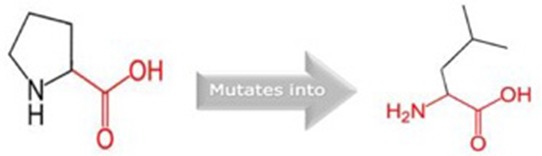	The mutant residue is bigger than the wild-type residue. The wild-type residue is a proline. Prolines are known to be very rigid and therefore induce a special backbone conformation which might be required at this position. The mutation can disturb this special conformation. Also, the mutated residue is located in a domain that is important for the main activity of the protein. The residue is located on the surface of the protein, mutation of this residue can disturb interactions with other molecules or other parts of the protein.

It is worthy of note that most of these phytochemicals failed the *in silico* drug-likeness test and showed poor gastrointestinal absorption (Table 6). However, this is not unusual of anticancer phytochemicals including polyphenols such as curcumin and green tea polyphenols; hence, it has been suggested that bioavailability of a compound cannot be accurately predicted (63). For many phytochemicals in this class, the uptake and efflux transporters at the epithelial cell surface also play a critical role in their bioavailability. One of such transporters that is relevant to cancer prevention and treatment is P-glycoprotein (64). Although some of these compounds are also P-glycoprotein substrates and this could eventually reduce their bioavailability, the coadministration of the P-glycoprotein inhibitors, such as erythromycin or clarithromycin, to patients receiving such P-glycoprotein substrate drugs have been noted to increase in their plasma and tissue concentrations (65). P-glycoprotein inhibitors have been employed as adjuncts in cancer chemotherapy, but their use in routine clinical practice is not approved due, in part, to inhibition of the CYP P-450 drug metabolizing enzyme (64). Our data also identified some of these compounds that do not inhibit CYP P-450 (Table 7). It is worthy of note that the Neochromes, although, are the substrate for P-glycoprotein, does not inhibit any of the CYP P-450 reported.

**Table 6:** Druglikeness properties of anti- GUCY1A2 phytochemicals.

**Compound name**	**Lipinski violations**	**Ghose violations**	**Veber violations**	**Egan violations**	**Muegge violations**	**Bioavailability Score**	**Brenk alerts**	**Leadlikeness violations**	**Synthetic accessibility**
(8′R)-neochrome	2	4	0	1	2	0.17	2	3	7.69
(8′S)-neochrome	2	4	0	1	2	0.17	2	3	7.69
22-epicalamistrin	2	2	1	0	2	0.17	2	3	4.73
3-beta-O-(E)-feruloylbetulin	2	4	0	1	2	0.17	2	2	7.82
3-hydroxy-6′-desmethyl-9-O-methylthalifaboramine	1	3	0	0	1	0.55	0	1	7.27
Aculeatin_A	0	2	1	1	1	0.55	0	3	6.82
Annoglaucin	1	4	1	1	3	0.55	0	3	7.76
Bullatetrocin	1	4	1	1	3	0.55	0	3	7.63
cis-3-O-p-Hydroxycinnamoyl_Ursolic_Acid	2	4	0	1	2	0.56	2	2	6.82
Desacetyluvaricin	2	4	1	1	3	0.17	0	3	7.39
Foveoglin_A	1	3	0	1	2	0.55	2	3	7.37
Foveoglin_B	1	3	0	1	2	0.55	2	3	7.37
Longimicins_A	1	4	1	1	3	0.55	0	3	7.56
Melianin_B	2	4	0	1	2	0.17	2	3	7.49
Melianin_C	2	4	0	1	2	0.17	2	3	6.61
Meliavolkinin	1	4	0	1	1	0.55	2	2	6.63
Muricatetrocin_C	1	4	1	1	2	0.55	0	3	7.34
Rollidecin_A	1	4	1	1	3	0.55	0	3	7.76
Silvestrol	2	3	2	1	3	0.17	0	2	6.6
Vinblastine	2	3	1	1	4	0.17	2	3	9.65

**Table 7:** Pharmacokinetics properties of anti- GUCY1A2 phytochemicals.

**Compound name**	**GI absorption**	**BBB permeant**	**P-glycoprotein substrate**	**CYP1A2 inhibitor**	**CYP2C19 inhibitor**	**CYP2C9 inhibitor**	**CYP2D6 inhibitor**	**CYP3A4 inhibitor**	**log Kp (cm/s)**
(8′R)-neochrome	Low	No	Yes	No	No	No	No	No	−3.24
(8′S)-neochrome	Low	No	Yes	No	No	No	No	No	−3.24
22-epicalamistrin	High	No	Yes	No	No	Yes	No	No	−1.04
3-beta-O-(E)-feruloylbetulin	Low	No	Yes	No	No	Yes	No	No	−2.78
3-hydroxy-6′-desmethyl-9-O-methylthalifaboramine	High	No	Yes	No	No	Yes	No	No	−8.03
Aculeatin_A	High	No	No	No	No	No	Yes	Yes	−3.69
Annoglaucin	Low	No	No	No	No	No	No	Yes	−4.65
Bullatetrocin	Low	No	No	No	No	No	No	Yes	−4.26
cis-3-O-p-Hydroxycinnamoyl_Ursolic_Acid	Low	No	No	No	No	No	No	No	−3.13
Desacetyluvaricin	Low	No	Yes	No	No	No	No	Yes	−2.03
Foveoglin_A	Low	No	Yes	No	No	No	No	No	−6.18
Foveoglin_B	Low	No	Yes	No	No	No	No	No	−6.18
Longimicins_A	Low	No	No	No	No	No	No	Yes	−3.21
Melianin_B	Low	No	Yes	No	No	No	No	Yes	−5.75
Melianin_C	Low	No	Yes	No	No	Yes	No	Yes	−5.13
Meliavolkinin	Low	No	Yes	No	No	No	No	No	−5.61
Muricatetrocin_C	Low	No	No	No	No	No	No	Yes	−3.71
Rollidecin_A	Low	No	No	No	No	No	No	Yes	−4.4
Silvestrol	Low	No	Yes	No	No	No	No	Yes	−9.13
Vinblastine	Low	No	Yes	No	No	No	No	Yes	−8.49

In conclusion, while these compounds may also have other targets in inducing cytotoxicity in prostate cancer, their ability to inhibit sGCα makes them more useful in addressing a complex disease like CRPC, rather than the usual “one gene, one target, one disease” approach which has limited the success of most anticancer drugs (66). The results of this investigation, therefore, suggest Neochrome a putative lead and possible nutraceutical in the treatment of CRPC.

## Data Availability

Data generated from this study are contained in the manuscript and Supplementary File.

## Author Contributions

SR, OR, and AS conceived and designed the approach and methodology. Data Analysis and interpretation was carried out by SR, OR, and PJ. JO and EI contributed to the writing of the manuscript. All authors reviewed the manuscript.

### Conflict of Interest Statement

The authors declare that the research was conducted in the absence of any commercial or financial relationships that could be construed as a potential conflict of interest.

## Supplementary Material

Table S1 List of most significant differently expressed genes.
